# A Selective Neutraligand for CXCL12/SDF-1α With Beneficial Regulatory Functions in MRL/Lpr Lupus Prone Mice

**DOI:** 10.3389/fphar.2021.752194

**Published:** 2021-10-21

**Authors:** Nicolas Schall, François Daubeuf, Claire Marsol, Patrick Gizzi, Nelly Frossard, Dominique Bonnet, Jean-Luc Galzi, Sylviane Muller

**Affiliations:** ^1^ CNRS UMR7242, Biotechnology and Cell Signaling, Ecole Supérieure de Biotechnologie de Strasbourg, Strasbourg University/Strasbourg Drug Discovery and Development Institute (IMS), Strasbourg, France; ^2^ CNRS UMR7200, Laboratoire d’innovation Thérapeutique, Faculté de Pharmacie, Strasbourg University/Strasbourg Drug Discovery and Development Institute (IMS), Strasbourg, France; ^3^ CNRS UMS3286, Plate-forme de Chimie Biologique Intégrative de Strasbourg, Strasbourg University/ Strasbourg Drug Discovery and Development Institute (IMS), Strasbourg, France; ^4^ Fédération Hospitalo-Universitaire (FHU) OMICARE, Fédération de Médecine Translationnelle de Strasbourg (FMTS), Strasbourg University, Strasbourg, France; ^5^ University of Strasbourg Institute for Advanced Study (USIAS), Strasbourg, France

**Keywords:** inflammation, MRL/lpr mouse model, neutraligand, CXCL12/CXCR4 axis, therapy

## Abstract

Dysregulation of CXCL12/SDF-1-CXCR4/CD184 signaling is associated with inflammatory diseases and notably with systemic lupus erythematosus. Issued from the lead molecule chalcone-4, the first neutraligand of the CXCL12 chemokine, LIT-927 was recently described as a potent analogue with improved solubility and stability. We aimed to investigate the capacity of LIT-927 to correct immune alterations in lupus-prone MRL/lpr mice and to explore the mechanism of action implemented by this small molecule in this model. We found that in contrast to AMD3100, an antagonist of CXCR4 and agonist of CXCR7, LIT-927 reduces the excessive number of several B/T lymphocyte subsets occurring in the blood of sick MRL/lpr mice (including CD3^+^/CD4^-^/CD8^-^/B220^+^ double negative T cells). *In vitro*, LIT-927 downregulated the overexpression of several activation markers on splenic MRL/lpr lymphocytes. It exerted effects on the CXCR4 pathway in MRL/lpr CD4^+^ T spleen cells. The results underline the importance of the CXCL12/CXCR4 axis in lupus pathophysiology. They indicate that neutralizing CXCL12 by the neutraligand LIT-927 can attenuate hyperactive lymphocytes in lupus. This mode of intervention might represent a novel strategy to control a common pathophysiological mechanism occurring in inflammatory diseases.

## Introduction

C-X-C chemokine 12 (CXCL12), also known as stromal cell-derived factor-1 alpha (SDF-1α), is central in the regulation of migration, proliferation, and differentiation of hematopoietic cells. It notably plays important roles in the homeostatic and inflammatory traffic of leukocytes to specific niches in lymphoid organs and inflammatory sites (Pawig et al., 2015; Janssens et al., 2018). There are at least six different isoforms of CXCL12, which are expressed in different peripheral tissues. The main receptor of CXCL12 is C-X-C chemokine receptor type 4 (CXCR4), also known as fusin or CD184 (Kawaguchi et al., 2019), a member of the superfamily of G protein-coupled receptors, broadly expressed at the surface of many leukocytes, including T cells, B cells, monocytes, neutrophils and dendritic cells (Nanki et al., 2000). A second receptor for CXCL12 is CXCR7/ACKR3/GPR159, which also binds, albeit with lower affinity, to CXCL11/interferon-inducible T-cell alpha chemoattractant, and might play a role in many immunomodulatory functions (Sánchez-Martín et al., 2013).

Because chemokines and especially CXCL12 can facilitate decisive crosstalk between B and T cells, notably by recruiting these immune cells in lymphoid and non-lymphoid tissues and organs (Minami et al., 2017), the CXCL12/CXCR4 axis received particular attention in autoimmunity (Karin, 2010; Janssens et al., 2018). Some studies pioneered this field to some extent and notably pointed out the importance of the CXCL12/CXCR4 pathway in systemic lupus erythematosus (SLE) physiopathology (Henneken et al., 2005; Chong and Mohan, 2009; Zhao et al., 2017; Barrera-Vargas et al., 2018). SLE is a complex polygenic autoimmune disease that can potentially affect persons of all ages and ethnic groups. More than 90% of new patients presenting with SLE are women in the childbearing age. The disease is associated with a chronic activation of the immune system, leading to patients with a complex array of immune abnormalities, which perpetuate in a self-sustaining process over years. B and T cell dysfunctions are central in lupus (Moulton et al., 2017). They result from intrinsic cell defects and also from aberrant signaling by regulating/suppressive immune cells with subsequent alteration in antigen presentation to T cells, production of cytokines (including interferons, IL-6, and IL-10), B cell signaling and differentiation, and autoantibody production. The expression of a number of co-stimulatory molecules, such as CTLA-4/CD152, and soluble mediators, such as BAFF/BLyS, a critical survival factor for transitional and mature B cells, is also dysregulated in lupus (Zhang and Vignali, 2016). In the same vein, expression levels of CXCL12 and its two cognate receptors CXCR4 and CXCR7 have been found to be altered in SLE patients and murine models of lupus. Although independent investigations reported somewhat divergent results according to the origin of patients and probably also in connection with their treatments, in general the serum levels of CXCL12 and the expression of CXCL12 and CXCR4 in the kidneys from patients with SLE were found to be enhanced as compared to control groups, and correlated with the activity of the disease (Robak et al., 2007; Wang et al., 2010; Launay et al., 2013; Hanaoka et al., 2015; Barrera-Vargas et al., 2018), CXCR4 was found to be overexpressed on both circulating CD19^+^ B cells and CD4^+^ T-cells. Some other studies, however, showed that expression of CXCR4 remained unchanged (Amoura et al., 2003) or was decreased in all memory T cell subtypes from lupus patients compared to controls (Fritsch et al., 2006; Chong and Mohan, 2009; Biajoux et al., 2012). CXCR4 was also found to be expressed at similar levels by antibody-secreting cells from SLE patients and healthy controls (de la Varga Martínez et al., 2017). Hyperexpression of CXCR4 on several peripheral blood leukocyte subsets was demonstrated in murine lupus models with active nephritis, namely (NZBxNZW)F1, B6.Sle1Yaa, BXSB, and MRL/lpr (Murphy Roths large/lymphoproliferation) mice (Balabanian et al., 2003; Wang et al., 2009; Cheng et al., 2018). This up-regulated expression of CXCR4 was claimed to be responsible for prolonged B cell lifespan and augmented B cell chemotaxis. Analyses of kidneys of nephritic MRL/lpr mice revealed elevated levels of CXCL12 (Wang et al., 2009).

Altogether, these data illustrate that in lupus, major alterations occur in the CXCL12/CXCR4 axis, and suggest that measuring the expression levels of CXCL12 and/or CXCR4 could be useful to follow the activity status of patients. Targeting the CXCL12/CXCR4 pathway might accordingly represent an important way for therapeutic applications. Gaining better insight into biological effects of CXCR4 signaling and underlying cellular and molecular mechanisms is thus of first clinical importance. In this context, here, we examined the potential of a novel molecule called LIT-927, which binds to CXCL12 but not its two cognate receptors CXCR4 and CXCR7 (Regenass et al., 2018). This small molecule that was derived from chalcone-4 (Hachet-Haas et al., 2008; Daubeuf et al., 2013) and its highly soluble prodrug derivative chalcone-4-P (Gasparik et al., 2012) therefore differs radically from known CXCR4 antagonists such as AMD3100, a bicyclam molecule that reversibly antagonizes CXCR4 and is an allosteric agonist of CXCR7 (De Clercq, 2005; Kalatskaya et al., 2009), peptide LY2510924, peptide E5 or clobenpropit and IT1t small molecules (Guo et al., 2017; Smith et al., 2019). LIT-927 also differs from CTCE-9908, a 17 residues-long peptide analogue mimicking CXCL12 (Kim et al., 2008; Wang et al., 2009) and EPI-X4, a 16 residues-long endogenous peptide of albumin (Zirafi et al., 2015; Zirafi et al., 2016), which acts on CXCR4/CXCR7 directly. LIT-927 behaves as a “neutraligand” that interacts directly with the chemokine CXCL12 and could block it before it crosses the blood brain barrier known to be leaky in neuropsychiatric forms of lupus (Jeltsch-David and Muller, 2014; Nikolopoulos et al., 2019) or before it reaches its targets in the kidneys, for example. In this report, we examined the in vivo effect of LIT-927 in diseased MRL/lpr lupus-prone mice and found that the CXCL12 neutraligand LIT-927 significantly reduces peripheral hypercellularity that characterizes this mouse model. We show further that LIT-927 modulates the activation status of effector and regulatory splenic lymphocyte subsets. We identified the post-CXCR4 molecular pathway that is probed by LIT-927 in splenic cells.

## Materials and Methods

### Mice

Female MRL/lpr mice were purchased from Charles River-France or bred and housed in our colony facilities. Some experiments used outbred CD-1 and inbred C57BL/6 mice, which were purchased from Charles River-France. CBA/J mice were purchased from Harlan (Gannat, France). Animal protocols were carried out with the approval of the local Institutional Animal Care and Use Committee (CREMEAS, Strasbourg, France) and the French Ministère de l’Enseignement Supérieur de la recherche et de l’innovation (APAFIS #3670-201601201121159 and #20139-2019032909253153). According to these agreements, and taking into account the best European practices in the field (3-R rules), we took the necessary measures to avoid pain and minimize the distress and useless suffering of mice during the time of experiment and killing process. Animals were maintained under controlled environmental conditions (20 ± 2°C) in conventional husbandry and 12 h/12 h light-dark cycle (lighting 7:00 a.m.–7:00 p.m.) was imposed. Mice were kept in large polycarbonate cages with 8–10 mice per cage with bedding made from spruce wood chips (Safe) and enriched with play tunnels which were changed every week.

### LIT-927, Chalcone-4-P and Other Test Molecules

**FIGURE 1:**
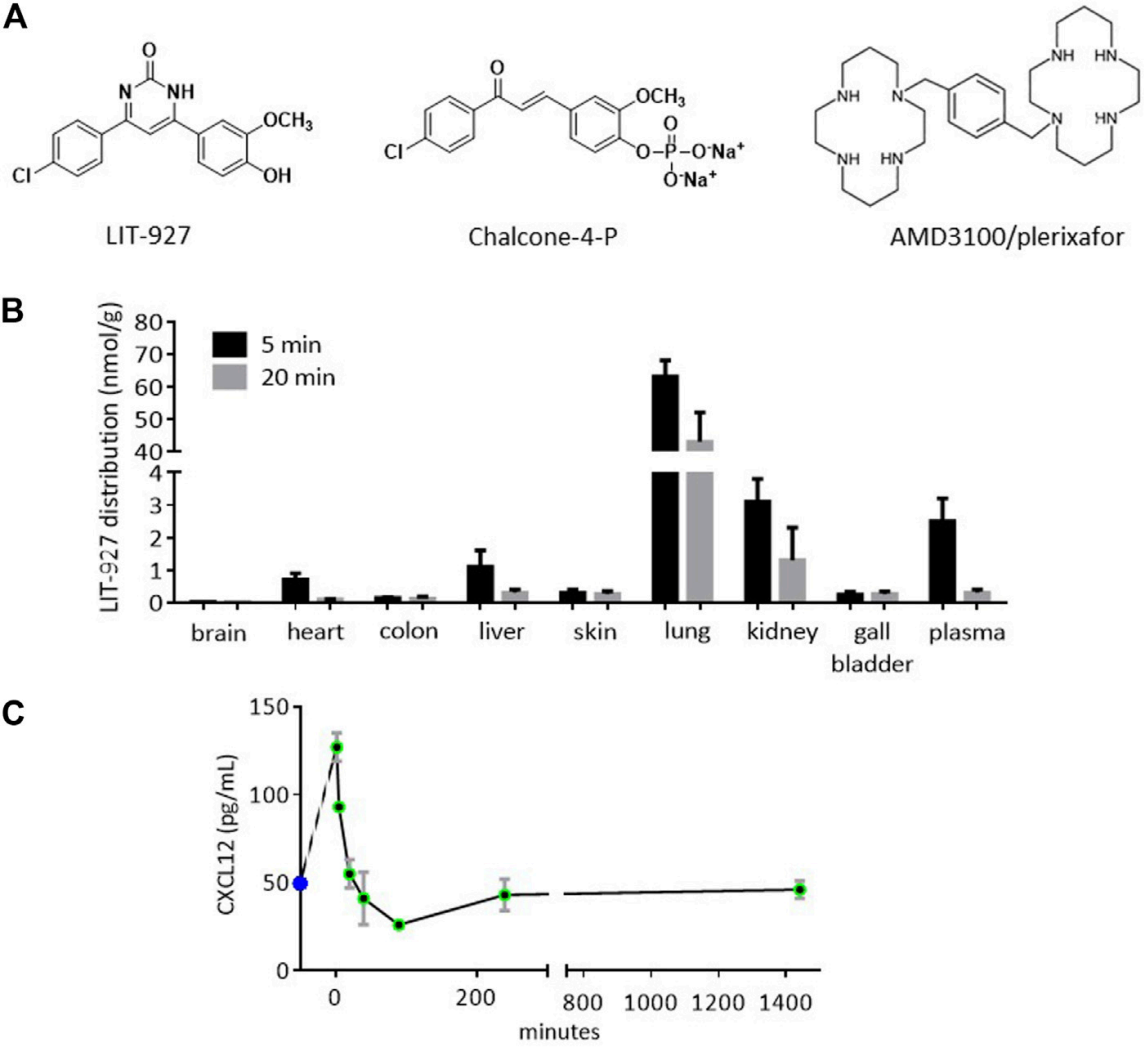
Structure and biodistribution of LIT-927, and effect on the plasmatic CXCL12 level after intravenous administration into healthy mice. **(A)** Chemical structures of CXCL12 neutraligand (LIT-927), prodrug of CXCL12 neutraligand (chalcone-4-P) and CXCR4 antagonist (AMD3100). **(B)** Biodistribution in organs and tissues of LIT-927 (1 mg/kg) after iv administration in CD-1 normal mice (*n* = 3). The results are expressed as nmol/g except for gall bladder (nmol in the total bladder) and plasma (nmol/L). **(C)** CXCL12 concentration in pg/mL measured by ELISA in the plasma of CD-1 normal mice (*n* = 3) after iv administration of LIT-927 (1 mg/kg). The results are means ± SD.

LIT-927 (initially described as the pyrimidinone 57 compound; MW 300.27 g/mol: Figure 1A) was synthesized, purified and characterized as described (Regenass et al., 2018). Chalcone-4-P (MW 395.99 g/mol) was produced and characterized as described (Gasparik et al., 2012). AMD3100/Plerixafor (MW 794.5) was purchased from Sigma (product #A 5602). The maximal solubility of LIT-927, chalcone-4-P and AMD3100 is 36.4 µM in phosphate-buffered saline (PBS), >30 mM in 2-[4-(2-hydroxyethyl)piperazin-1-yl]ethanesulfonic acid (HEPES), and 28 mM in water, respectively (Gasparik et al., 2012; Regenass et al., 2018). (2-hydroxypropyl)-β-cyclodextrin (HPβCD; Sigma, product #C0926) was used as an excipient to both increase the solubility of LIT-927, which is > 3 mM in PBS/10% (w/v) HPβCD, and improve its bioavailability as it allows easier diffusion of lipophilic compounds across biological membranes. Molecules classically given to patients with inflammatory diseases, namely cyclophosphamide/Endoxan, prednisolone/Cortancyl, hydroxychloroquine/Plaquenil, FTY720/Fingolimod, amethopterin/Methotrexate, azathioprine/Imurel, mycophenolate mofetil/Cellcept), and sirolimus/Rapamune were administrable, commercially available in pharmaceutical forms. Risdronate/Actonel, a powerful bone resorption inhibitor that belongs to the bisphosphonate category, and desloratadine/Aerius, an anti-histamine compound, were added as controls.

### In Vivo Pharmacokinetics and Biodistribution of LIT-927 in Mice

An in vivo PK and biodistribution study was performed using 6-week-old CD-1 mice (Cox et al., 2002). LIT-927 was administered intravenously (iv; caudal vein route; 2 mL/kg per injection) at a dose of 1 mg/kg. Blood was drawn by intracardiac puncture at several time points (2, 5, 20, 40, 90 min, 4 and 24 h post-treatment; 3 mice per time point). Blood samples collected in ethylenediamine tetraacetic acid (EDTA)-coated tubes were centrifuged at 10,000 g for 2 min at 4°C to collect plasma, which was frozen in liquid nitrogen and then stored at −80°C until analysis. Mice were perfused with 20 mL of PBS-EDTA 3 mM solution and various organs and tissues (blood, brain, kidney, lung, liver, heart, colon and skin) were collected post-mortem and immediately frozen at −80°C. Plasma samples were mixed with acetonitrile (ratio 2.5:1) in order to precipitate proteins. Tissues were grinded in water and 2 volumes of acetonitrile were added to extract the compound. Samples were vortex-stirred for 5 min, sonicated for 1 min and thereafter centrifuged at 15,000 g during 5 min at 4°C to collect precipitates. Supernatants were transferred into a microplate and were analyzed by liquid chromatography tandem mass spectrometry (LC-MS/MS) using an ultra-performance liquid chromatography system coupled to a triple quadrupole Shimadzu LC-MS 8030 mass spectrometer. For all kinds of samples, standard ranges of concentrations were analyzed in the same series of injections. Separations were carried out at 40°C using a 2.6 µm C18 Kinetex column (50 × 2.1 mm) from Phenomenex. The mobile phase flow rate was set at 0.5 mL/min and the following program was applied for the elution: 0 min, 5% B; 0–1.2 min, 5–95% B; 1.2–1.4 min, 95% B; 1.4–1.42 min, 95–5% B and 1.42–2.8 min, 5% B (solvent A: 0.05% formic acid in water; solvent B: acetonitrile). Injection volume was 2 µL. The mass spectrometer was interfaced with the liquid chromatograph using an electrospray ion source. The MS/MS conditions were optimized as follows: capillary voltage, 4.5 kV; nitrogen nebulizing gas flow, 1.5 L/min and drying gas flow, 15 mL/min; block heater and desolvation line temperature, respectively 400 and 250°C. The collision gas used was argon at 230 kPa. Analysis was performed in multiple reaction-monitoring mode. The transition was m/z 329.0 → 313.9, 285.9.

### Measurement of the Plasma Level of CXCL12 After Intravenous Administration Into Mice

The ELISA kit for mouse CXCL12 (R&D Systems, ref. DY460; kit range: 46–3,000 pg/mL; sensitivity: 23 pg/mL) was used according to the manufacturer’s instructions.

### In Vivo Biological Activity of LIT-927 in MRL/Lpr Lupus Mice

LIT-927 in HPβCD and all other molecules were suspended in 9‰ NaCl. Groups of 11–13 week-old MRL/lpr mice received a single iv administration of these molecules (retro-orbital route; 100 µL per injection) at a dose of 4 mg/kg for all molecules except LIT-927 (3.9 mg/kg), chalcone-4-P (5.5 mg/kg), AMD3100 (5 mg/kg) and Sirolimus (2 mg/kg). In addition, the pharmaceutical drugs contain several excipients and additives that are listed in the notices provided by the manufacturers. The control group of mice (“untreated”) received 100 µL of vehicle only. Five days later, mice were bled and their spleen was collected.

### Peripheral Hypercellularity Measurements

The test was as described (Schall et al., 2012; Briand et al., 2014). Briefly, red blood cells collected from individual mice of different groups were lysed using EasyLyse reagent (DAKO, ref. S2364) according to the manufacturer’s protocol. After centrifugation, white blood cells (WBCs) were stained with acridine orange/propidium iodide and counted using a LUNA FL apparatus (Logos Biosystems, Annandale, United States). The number of living WBCs in each group was compared to the one counted in the control group and the decrease of cell numbers was calculated. To avoid any bias due to groups of mice, the results mixed from several independent experiments are presented. The results are expressed as the mean decrease of peripheral WBC percentage ± SEM. Statistical differences were determined using the unpaired t test for groups containing ≥ 8 mice. Statistical results were calculated using the GraphPad Prism software.

### FACS Analysis

To measure the effect of LIT-927 on activation markers and other characteristic markers expressed by CD4^+^ T cells, CD8^+^ T cells and CD19^+^ B cells of MRL/lpr and C57BL/6 mice used as healthy controls, the following conjugated antibodies were used: CD19-FITC, CD154/CD40 ligand (L)-PE, CD25-PerCP-Cy5.5, CD3-PE-Cy7, CD45-SuperBright436, B220-eFluor506, CD40-SuperBright600, CD86-SuperBright702, CD184/CXCR4-APC, CD4-AlexaFluor700, CD8a-APC-Cy7 (all from ThermoFisher Scientific) and FOXP3-APC (Invitrogen). Double-negative (DN) T cells that typically expand in lupus patients and MRL/lpr mice display a CD3^+^CD4^−^CD8^-^B220^+^ phenotype. FOXP3 intracellular staining was performed using the eBioscience Foxp3/Transcription factor staining buffer set (ref. 00–5523-00) according to the manufacturer’s instructions. Fluorescence was measured with an Attune NxT cytometer, and flow data were analyzed using FlowJo software (FlowJo, LLC).

### Western Blot Analyses

Spleens were collected from four 7-week-old MRL/lpr and CBA/J mice and cut into small pieces. At this age, MRL/lpr mice develop no clinical sign of disease and possess no antibodies to native DNA in their serum. Spleens (0.2 g of tissue) were homogenized in 3.5 mL of boiling lysis buffer (10 mM Tris pH 7.4, 1 mM sodium orthovanadate, 1% v/v SDS). An equal volume of 2x concentrated electrophoresis sample buffer (125 mM Tris pH 6.8, 4% v/v SDS, 10% v/v glycerol, 0.006% v/v bromophenol blue, 2% v/v β-mercaptoethanol) was added and samples were frozen at −80°C until use. Western immunoblotting analysis was performed using the PowerBlot system developed by BD-Biosciences. Denaturing electrophoresis (SDS-PAGE; 4–15% gradient, 13 × 10 cm, 0.5 cm thick) was used to analyze the proteins (IPG well comb; Biorad). Protein samples (200 µg/gel) were loaded in one large well across the entire width of the gel. Each gel was run for 1.5 h at 150 V and then transferred to Immobilon-P membrane (Millipore) for 2 h at 200 mA using a wet electrophoretic transfer apparatus TE series from Hoefer (Amersham Pharmacia Biotech). After transfer, the membrane was dried, dipped in methanol and saturated for 1 h with blocking buffer (LI-COR, Lincoln, NE). The membrane was then clamped with a western blotting manifold that isolates 40 channels across the membrane. In each channel, a complex mouse monoclonal antibody cocktail was added and allowed to react for 1 h at 37°C. The blot was removed from the manifold, washed and incubated for 30 min at 37°C with a secondary anti-mouse Ig conjugated to Alexa-680 fluorescent dye (Molecular Probes). The membrane was washed, dried and scanned using the Odyssey Infrared Imaging System (LI-COR). Molecular weight (molar mass) standards were composed of an antibody panel added to lane 40 of each gel. Each sample was run in triplicate in three independent experiments and more than 800 selected monoclonal antibodies were tested (BD-Biosciences). To normalize the gels, the raw quantity of a spot was divided by the total intensity value of all valid spots of each image multiplied by 1,000,000. The results were analyzed using a 3 × 3 matrix comparison analysis method. For example, each run of sample 1 (CBA/J cells) was compared to runs 1, 2 and 3 of sample 2 (MRL/lpr cells). In order to avoid any over-interpretation of the results, highly significant differences only have been highlighted, namely changes greater than 1.25-fold in all 9 comparisons in the 3 × 3 matrix comparison method.

To study the effect of LIT-927 on the post-CXCR4 activation pathway, splenocytes were routinely isolated on nylon membrane (70 µm) and pooled from two 11–13 week-old mice. CD4^+^ T cells were enriched using Miltenyi Biotech CD4^+^ T cells isolation kit (ref. 130–104–454) and immediately cultured at 37°C, 5% CO2 in RPMI 1640 medium supplemented with 10% v/v FCS, 10 mg/mL gentamycin, 10 mM HEPES and 0.05 mM β-mercaptoethanol (all from Lonza BioWhittaker) at a concentration of 5 × 10^6^ cells/mL. They were then exposed to 0 or 100 ng/mL of recombinant CXCL12 (R&D Systems) for 5 min at 37°C in the presence or not of LIT-927 or AMD3100. Cells were collected and lysed with cold radioimmunoprecipitation assay buffer (RIPA) buffer (20 mM Tris-HCl pH 7.5, 150 mM NaCl, 1 mM Na_2_-EDTA, 1 mM EGTA/egtazic acid, 1% v/v NP-40) and the samples were prepared for SDS-PAGE. Following the transfer of proteins from the gels (4–20% gradient) onto nitrocellulose, the blots were probed with antibodies against total ERK1/2 (#9102), phosphorylated (P)-ERK1/2 (#9101), GSK3b (#9315), P-GSK3b (#9323), total AKT (#9272), P-AKT (#9271), P-β-CATENIN (#9561), MTOR (#2972) and P-MTOR (#2974), all from Cell Signaling Technologies, as well as against β-catenin (AF1329-SP, R&D Systems). Results are normalized with protein quantity for each sample using Biorad stain free technology.

### Statistics

Statistical analysis was performed using GraphPad Prism version 6 Student’s t test (parametric data) or Mann-Whitney U test (non-parametric data) used for two-group comparisons. For two-group comparison studies, we used unpaired t test when *n* ≥ 8 per group or Mann-Whitney test when *n* < 8 per group. One-way ANOVA with Bonferroni’s multiple comparison test was used for multiple group analysis. Any *p* value below 0.05 was considered significant. Experiments were repeated at least twice, and usually three or more times.

## Results

LIT-927 is the first locally and orally active CXCL12 chemokine-neutralizing molecule (neutraligand) shown to display anti-inflammatory effects in vivo, demonstrated in two murine models mimicking allergic airway hypereosinophilia (Regenass et al., 2018) and pulmonary arterial hypertension (Bordenave et al., 2020), respectively. The activity of CXCL12 neutraligands, and of LIT-927 in particular, has not been studied previously in the context of animal models of lupus and systemic autoimmunity in general. We explored the activity of LIT-927 in MRL/MpJ-Faslpr (MRL/lpr) mice that have a Fas mutation inducing spontaneous development of systemic autoimmune diseases and a short life span (half of MRL/lpr mice die by 5–6 months of age due in particular to lupus nephritis). In our studies, chalcone-4P and AMD3100 (Figure 1A) were used as control molecules.

### In vivo Biodistribution of LIT-927 After Intravenous Administration and Effect on the Plasma Level of CXCL12

Previous studies in animal models were performed in BALB/c mice or Wistar rats that received LIT-927 *via* the intranasal or intraperitoneal routes, respectively (Regenass et al., 2018; Bordenave et al., 2020). Since in our mouse model the molecules are given iv (Schall et al., 2012), we first tested the biodistribution of LIT-927 in this setting. Five minutes after injection, LIT-927 administered iv at a concentration of 1 mg/kg (2 mL/kg) in PBS/HPβCD primarily accumulated in the lungs (sustained) and kidneys (transient) of outbred CD-1 normal mice (Figure 1B and Supplementary Figure S1). Although less abundant, it was also found in the liver, heart, and skin of recipient mice. LIT-927 was present at very low level in the colon and was virtually absent in the brain of CD-1 mice. The maximal concentration of LIT-927 in urine was reached 90 min after injection (6.5 nmol/mL, equivalent to 2.1 μg/mL). No sign of any in vivo toxicity was recorded in CD-1 mice.

In vitro interaction of LIT-927 with CXCL12 has been previously demonstrated by showing its ability to inhibit the interaction between the chemokine and CXCR4 and by following the modification of fluorescence emission of tryptophan residue present in CXCL12 (Regenass et al., 2018). The evaluation by ELISA of circulating CXCL12 levels in the plasma of mice that received LIT-927 (1 mg/kg) *via* the iv route showed a sharp increase of the chemokine reaching 136 pg/mL at time 2 min, rapidly followed by an abrupt decrease to 24 pg/mL at 90 min and stabilization at a level of 50 pg/mL at 4 h that remained stable for at least 22 h (Figure 1C).

### Effects of LIT-927 on the Peripheral Cells in MRL/Lpr Lupus Mice

**FIGURE 2:**
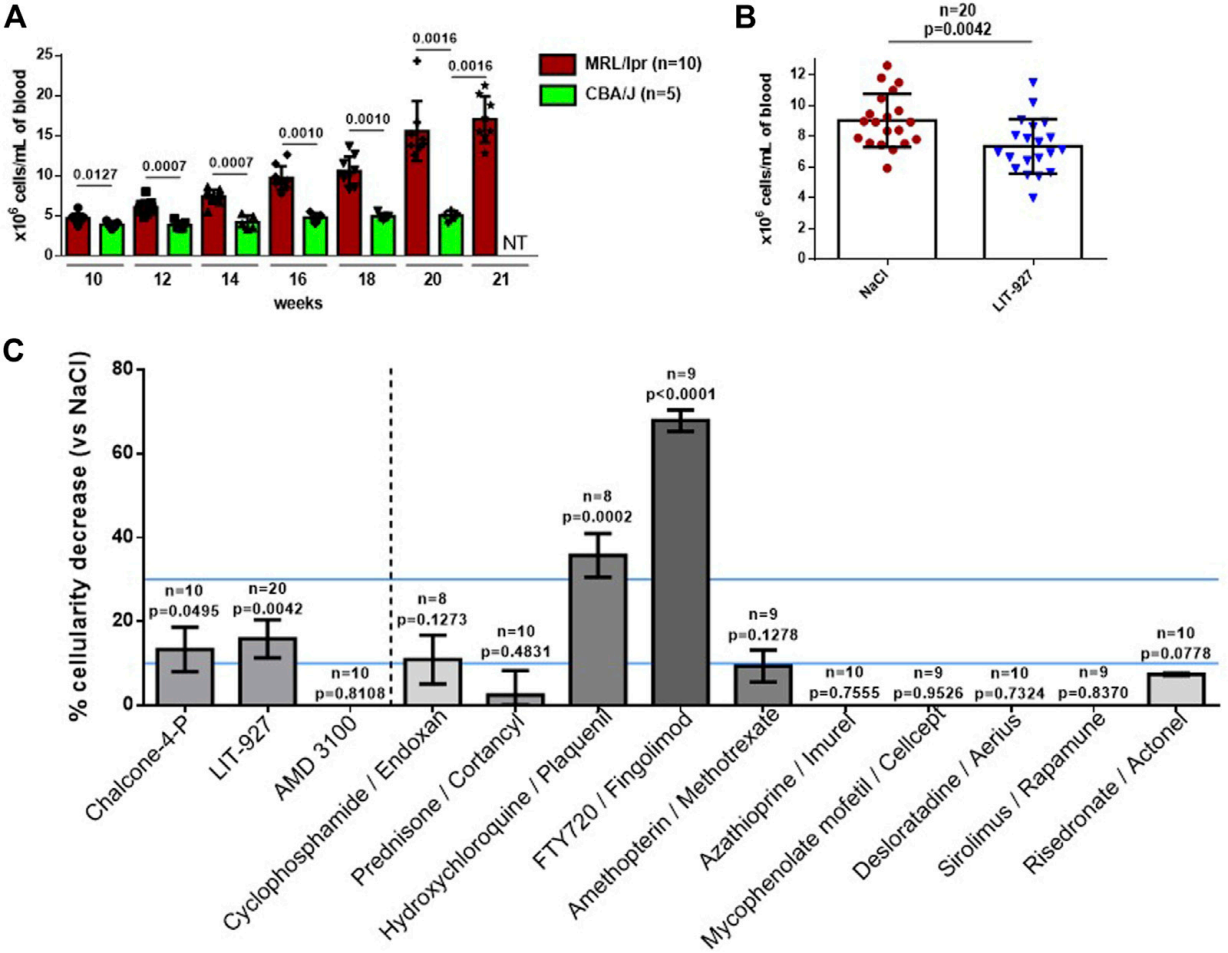
Effect of LIT-927 on the peripheral hypercellularity naturally-occurring in MRL/lpr mice. **(A)** Number of WBC in MRL/lpr mice of increasing age compared to healthy CBA/J mice. The number of WBCs/mL was evaluated by counting cells at day +5. Each symbol represents one individual mouse (n, number of mice/group). The results are means ± SD. p values were calculated from Mann-Whitney test. **(B)** Number of WBC in 12-week-old MRL/lpr mice that were either left untreated (NaCl group, *n* = 20) or that were treated with LIT-927 (*n* = 20). Each symbol represents one individual mouse. The results are means of WBCs ±SD. Statistical significance was assessed using the unpaired t-test. **(C)** Head-to-head comparison of the effect of LIT-927, chalcone-4-P, AMD3100 and control anti-inflammatory drugs on the peripheral hypercellularity occurring in MRL/lpr mice. Groups of 8–20 MRL/lpr mice (11–13 week-old) received a single administration of either LIT-927 in HPβCD at a dose of 3.9 mg/kg, or chalcone-4-P (5.5 mg/kg in saline), AMD3100 (5.0 mg/kg in saline) or indicated pharmacological molecules (each at 4.0 mg/kg in saline, except sirolimus given at 2 mg/kg for solubility reasons). The control group received vehicle only. Molecules were administrated intravenously. Each tested dose was determined in preliminary experiments. The results are the mean reduction of peripheral WBCs percentage ± SEM. Statistical significance was assessed using the unpaired t-test. An efficacy range of 10–30% cellularity decrease (determined arbitrarily) is pursued. Above this line (upper line), the tested molecule is considered as immunosuppressive and potentially toxic.

Having tested the properties of LIT-927 *in vitro* and its bioavailability and biodistribution in vivo, we next evaluated its ability to reduce lupus-related dysfunctions in vivo. To this end, we intravenously injected LIT-927, as well as chalcone 4-P and AMD3100, to separate groups of diseased MRL/lpr mice and 5 days later, we measured their capacity to lower abnormal peripheral hypercellularity that typically occurs in this strain of mice due to Fas mutation (Figure 2A). This assay was exploited in earlier studies with a therapeutic peptide and showed a perfect fit with long-lasting (8–12 months) protection experiments (Schall et al., 2012). As shown in Figures 2B,C, while chalcone-4-P used at a dose of 4.9 mg/kg was effective at the limit of statistical positivity (*p* = 0.0495, compared to mice left untreated) and AMD3100 showed no activity, LIT-927 used at 3.9 mg/kg was highly effective at reducing the excessive number of WBCs (*p* = 0.0042 for *n* = 20, compared to untreated mice; *p* = 0.0399 and 0.0540, respectively, in two independent experiments using 10 mice each). The mean cellularity dropped from 9.04 × 10^6^ cells/mL in mice that received saline alone to 7.35 × 10^6^ cells/mL in mice that received LIT-927 intravenously (basal level of WBC in non-autoimmune 12 week-old CBA/J mice = 4.35 × 10^6^ cells/mL; Figures 2A,B). This effect was dose-dependent; given at 1.8 mg/kg to 10 other mice, the effect was not statistically significant (data not shown). The decrease of peripheral hypercellularity affected CD4^+^, CD8^+^ and DN T cells as well as B cells (Supplementary Figure S2).

The effect of LIT-927 was compared in the same assay to that of molecules classically given to patients with inflammatory diseases, and SLE in particular. As shown in Figure 2, while some molecules such as cyclophosphamide, prednisone, amethopterin, azathioprine, mycophenolate mofetil, sirolimus and control compounds desloratadine and risedronate had no significant effect in MRL/lpr mice on peripheral hypercellularity, two molecules displayed impressive potency at reducing the level of WBCs (above the upper blue line). This was hydroxychloroquine, used to treat the symptoms of SLE and rheumatoid arthritis (RA) and to prevent and treat malaria, that reduced cellularity to 4.64 × 10^6^ cells/mL in average (*p* = 0.0002), and FTY720, used to treat relapsing forms of multiple sclerosis, that reduced cellularity to 2.43 × 10^6^ cells/mL in average (*p* < 0.0001). These two molecules, however, may have deleterious effects that preclude their use in certain patients. Depending on the daily dose given to patients and the duration of administration (often for years in the case of autoimmune diseases), the main side effects of hydroxychloroquine are gastrointestinal upset, skin rash, headache, ocular toxicity, and cardiac arrhythmia (due to elongation of QT interval) (Schrezenmeier and Dörner, 2020). FTY720 (used at 4.0 mg/kg in saline) can also show unwanted toxicity. In our assay, it dramatically decreased the level of WBCs in normal C57BL/6 mice (68.7% in MRL/lpr mice and 65.9% in C57BL/6 mice; not shown), an unexpected result that should alert us to the possibility that this compound is dangerous in certain conditions of use.

Altogether, these results indicate that CXCL12 neutraligand LIT-927 -but not the CXCR4 antagonist AMD3100-is able to reduce the excessive level of WBCs circulating in the peripheral blood of diseased MRL/lpr mice, while showing no safety concern. Throughout the experiment time, we did not observe any visible toxic effects in LIT-927-treated MRL/lpr mice, such as premature death, weight loss, or abnormal aspects of organs at autopsy, for example. No change of behavioral was recorded.

### In Vivo and *Ex Vivo* Effect of LIT-927 on MRL/Lpr Spleen Cells

**FIGURE 3:**
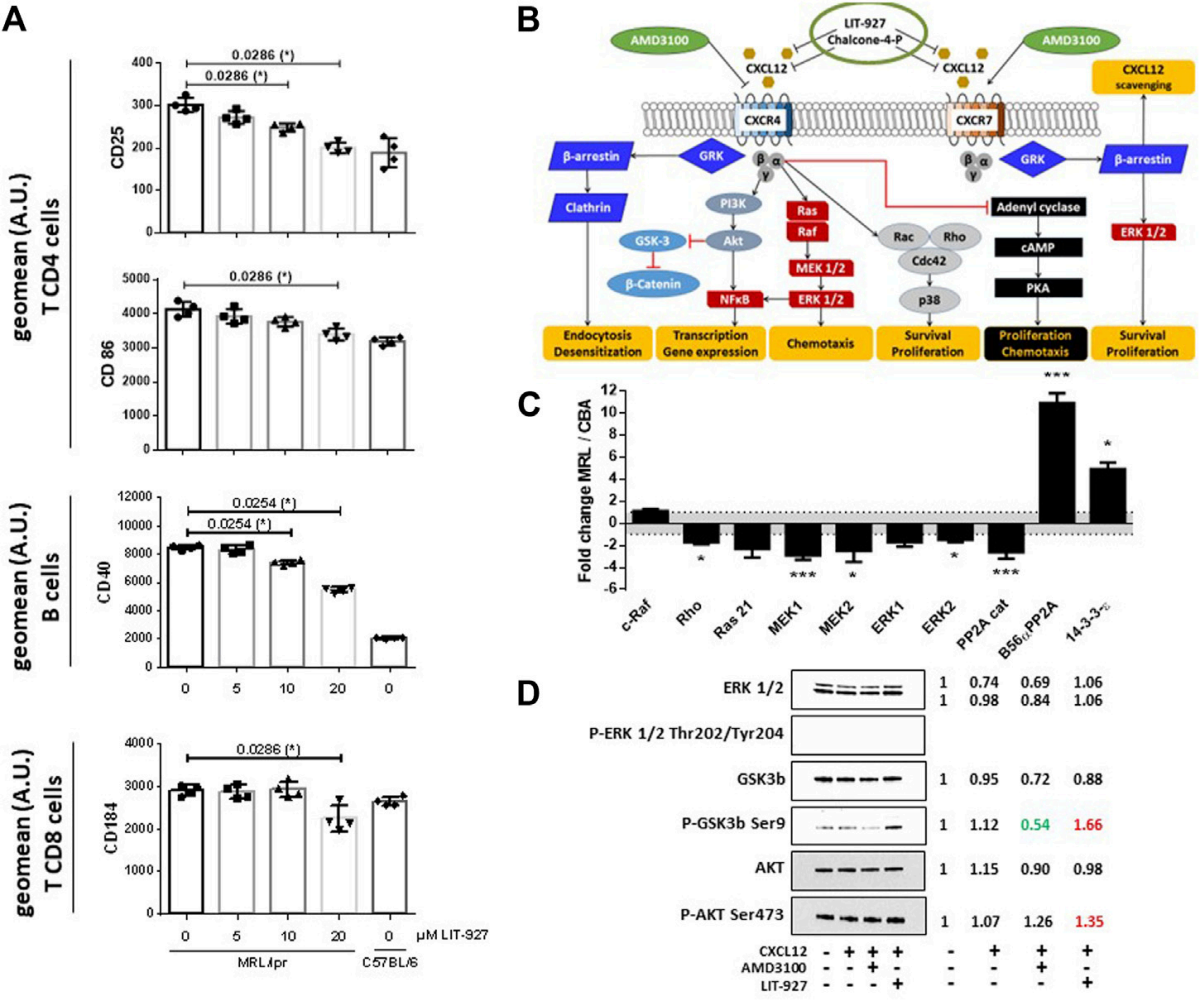
Effect of LIT-927 on the activation status and CXCR4 signaling pathway of MRL/lpr immune cells. **(A)** Repression of the expression of activity markers at the surface of MRL/lpr immune cells incubated with LIT-927. Unstimulated cells (2 × 10^6^ per well) sorted from the spleen of 11–13 week-old female MRL/lpr mice (2 mice per group; 2 independent experiments) were incubated or not with increasing concentrations of LIT-927 (0–20 µM) at 37°C for either 6 or 24 h and analyzed by flow cytometry (results shown at 24 h). Cells are defined as CD4^+^ or CD8^+^ T cells and CD19^+^ B cells. The results are expressed as the geometric mean fluorescence (geomean) intensity given in terms of arbitrary units (A.U.). **(B)** Schematic CXCL12/CXCR4 and CXCL12/CXCR7 intracellular signal transduction pathways and proposed sites of intervention of LIT-927, chalcone-4-P and AMD3100 (personal creating of the scheme). **(C)** Principal over- and under-expressed proteins involved in the CXCR4/CXCR7 intracellular signaling pathways in splenocytes from 7-week-old MRL/lpr mice. The variations of protein levels in MRL/lpr splenocytes are represented relative to the protein levels in splenocytes from CBA/J mice, after normalization of signals as described in the Method section. Each sample was run in triplicate in three different gels. Comparison was performed by the 3 × 3 matrix comparison method and error bars denote standard error. *p* values are represented (*p ≤ 0.05; **p ≤ 0.01; ***p ≤ 0.005). **(D)** CD4^+^ T cells pooled from the spleen of two 11–13 week-old MRL/lpr mice were incubated for 5 min in the presence or not of recombinant CXCL12, LIT-927 or AMD3100, as indicated. Cells were collected, lysed and subjected to SDS-PAGE using 4–20% gradient gels. After transfer to nitrocellulose membrane, the presence of a selected panel of proteins of the CXCL12-CXCR4 pathway was revealed by specific antibodies. The expression of indicated proteins in the four samples was normalized to the total relevant proteins using Biorad stain free technology. For each sample tested in the four conditions, the ratio expressed protein over the control (= no CXCL12 and no neutraligand) is indicated on the right.

From approximately 8 weeks of age, splenomegaly develops in MRL/lpr mice due to a massive increase of abnormal T lymphocyte subsets leading to a disruption of the normal splenic architecture. The effect of LIT-927 was thus also evaluated in the spleen of sick MRL/lpr mice. While it showed no significant effect on CD8^+^ and DN T cells or on B cells, a statistically significant effect was seen post-treatment on CD4^+^ T cells, the number of which was decreased compared to MRL/lpr mice that received HPβCD only (Supplementary Figure S2). Ex vivo, in a dose-response manner, LIT-927 down-regulated the overexpression of CD25 and CD86/B7-2 activation markers at the surface of splenic MRL/lpr CD4^+^ T cells, and CD40 at the surface of MRL/lpr B cells (Figure 3A). It decreased the expression of FOXP3 at the surface of MRL/lpr CD4^+^CD25^+^ and CD4^+^CD25^−^ cells, suggesting an effect on some mature regulatory T cells (Tregs) and Treg precursors (Supplementary Figure S3).

In this study, the expression of CD184/CXCR4 was not different on splenic MRL/lpr and C57BL/6 B cells (not shown). It was however upregulated on both MRL/lpr CD4^+^ T cells (*p* < 0.0001) and CD8^+^ T cells (*p* = 0.0109), and this overexpression of CXCR4 was significantly decreased at the surface of CD8^+^ T cells -but not CD4^+^ T cells (not shown)- upon LIT-927 treatment (Figure 3A; *p* = 0.0286).

### Effect of LIT-927 on the CXCR4 Signaling Pathway

**FIGURE 4:**
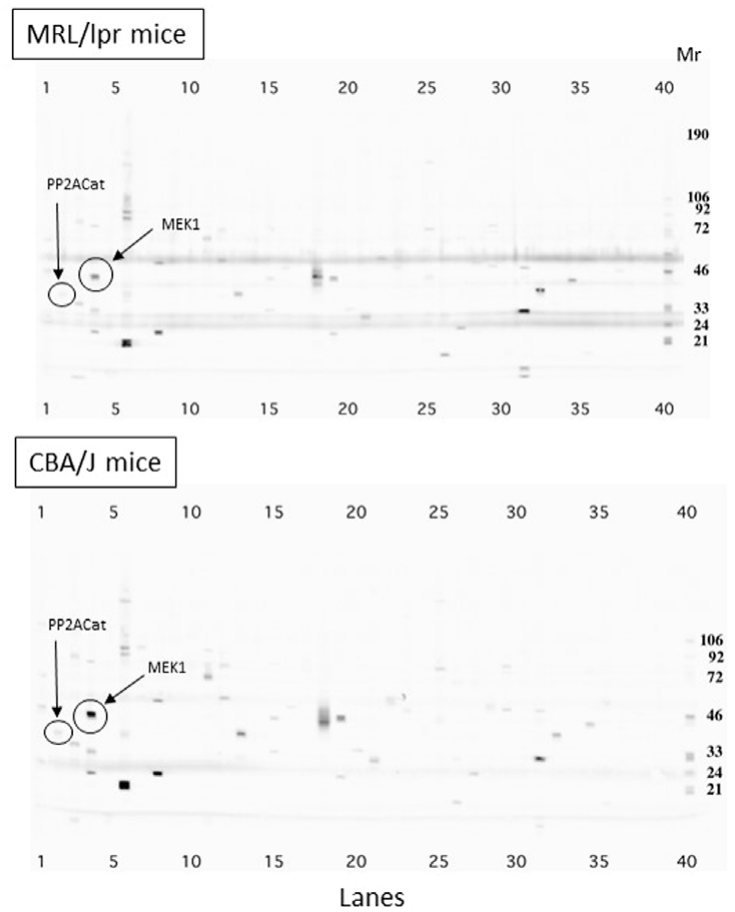
Comparison of protein expression in splenocytes from 7-week-old MRL/lpr and CBA/J mice. Proteins from spleen cells were separated by 4–15% gradient SDS-PAGE and identified using a panel of about 800 different monoclonal antibodies directed against cellular proteins. The differential expression in MRL/lpr mice and CBA/J mice of MAPK1/MEK1 protein is highlighted as an example. Mr markers are shown on the right (lane 40). Bands observed at 50 kD and 25 kD correspond to immunoglobulin heavy and light chains present in large amount in the splenocytes from lupus mice. Cat, catalytic.

Upon activation of CXCR4, several signaling cascades are activated (Figure 3B). As it was claimed that the CXCR4 pathway is deregulated in lupus (Wang et al., 2009, 2010; Biajoux et al., 2012; Launay et al., 2013; Zhao et al., 2017; Barrera-Vargas et al., 2018; Cheng et al., 2018), we examined if LIT-927 might correct some of these abnormalities. We first performed a series of western blot studies aimed at determining with specific antibodies which proteins of these signaling pathways are up- or down-regulated in MRL/lpr splenocytes compared to basal levels measured in normal mice and could serve as key markers to investigate the effect of LIT-927. Splenocyte proteins from 7-week-old asymptomatic MRL/lpr mice and naïve CBA/J mice were separated by gradient SDS-PAGE and identified with a panel of 800 different antibodies directed against a wide range of cellular proteins. The representation of at least 58 proteins involved in various cellular pathways such as apoptosis, cell cycle, signaling and differentiation was found to be up- or down-regulated in MRL/lpr splenocytes (Supplementary Table S1). Among them, proteins of the mitogen-activated protein kinase (MAPK)/extracellular-signal-regulated kinase (ERK) pathway were significantly less abundant than normal (Figure 3C and Figure 4; mean factor 1.7 ± 0.4; *p* = 0.011 for ERK1). Compared to CBA/J mice, the amount of MAPK1/MEK1 detected was significantly decreased in MRL/lpr mice (mean factor 2.9 ± 0.4; *p* ≤ 0.001). At a lower degree of significance, MAPK2/MEK2 and ERK2 were also under-represented in MRL/lpr mice (mean factors 2.5 ± 1.0 and 1.5 ± 0.2, respectively; *p* ≤ 0.05). Although we found no statistically significant difference in the levels of both Ras21 and Raf proteins, the amount of Rho, a member of the Ras superfamilly of GTPases, was significantly decreased (mean factor 1.7 ± 0.2; *p* ≤ 0.05). In relation with the MAPK/ERK signaling pathway, we also found that the abundance of the protein phosphatase 2A (PP2A) catalytic subunit was significantly decreased in MRL/lpr splenocytes (mean factor 2.6 ± 0.6; *p* = 0.002). However, the amount of the PP2A regulatory subunit B56α (mean factor 10.9 ± 0.9; *p* ≤ 0.001) and of PP1 (mean factor 1.7 ± 0.3; *p* = 0.05) was increased. One peculiarity of PP1/PP2A phosphatases is their ability to counteract a great number of kinases, controlling thereby most of the phosphoproteome of the cell. The amount of the protein 14-3-3ε, one of the seven isoforms of the 14-3-3 family involved in signal transduction by binding to phosphoserine-containing proteins, was also significantly increased by a mean factor 4.9 ± 0.6 (*p* ≤ 0.05).

The above results suggest that several proteins of the CXCL12-CXCR4 axis are aberrantly represented in the splenocytes of young, pre-diseased MRL/lpr mice, while they still display no visible symptoms at this early age. The next question was therefore whether LIT-927 corrects these defaults in MRL/lpr splenocytes. Several markers were tested by western blot, namely ERK1/2, glycogen synthase kinase 3 beta (GSK3b), AKT/protein kinase B, β-Catenin, mammalian target of rapamycin (MTOR), and their phosphorylated forms. The data showed first that incubating MRL/lpr CD4^+^ T cells with exogenous CXCL12 had little effect on the expression of these markers (Figure 3D and Supplementary Figure S4), likely indicating that these cells are already affected by endogenous CXCL12 in vivo. Second, while ERK1/2 was detected in MRL/lpr CD4^+^ T cells incubated or not with CXCL12 and with or without the neutraligand LIT-927 and of the CXCR4 antagonist AMD3100, the presence of P-ERK1/2 (Thr^202^/Tyr^204^) could not be visualized. Third, while the expression of GSK3b was not affected by LIT-927 or AMD3100, the level of P-GSK3b (Ser^9^) was decreased upon incubation with AMD3100 and increased upon incubation with LIT-927, indicating possible opposite effects of these two small molecules. The level of P-AKT (Ser^473^) was slightly increased in response to LIT-927 and AMD3100 (Figure 3D and Supplementary Figure S4). Collectively, these results thus show that LIT-927 exerted effects on the CXCR4 pathway (AKT-GSK3b markers) that is abnormally regulated in MRL/lpr CD4^+^ T spleen cells.

## Discussion

In the present study, we provide novel insights into the CXCL12/CXCR4 axis biology in lupus. We show that CXCR4 is overexpressed on splenic CD4^+^ and CD8^+^ T cells of diseased 11–13 week-old female mice while its level of expression remains equivalent at the surface of splenic B cell from normal C57BL/6 and MRL/lpr mice of the same age. The data obtained by western blot experiments performed with a panel of 800 different antibodies indicate further that in the splenocytes of pre-diseased MRL/lpr mice, the representation of several components of the MAPK/ERK signaling pathway, which is downstream of Ras/Raf, is dysregulated (under- or over-expressed) with regard to age-matched CBA/J mice used as controls. In humans, it has been shown earlier that compared to cells from normal individuals and from patients with RA, MEK-catalyzed ERK-1/2 phosphorylation activity was significantly decreased in peripheral blood mononuclear cells and CD4^+^ T cells from patients with SLE despite the fact that normal levels of ERK were found (Deng et al., 2001). This decrease of ERK activity was shown to be directly proportional to disease severity. It has been proposed that impaired ERK signaling pathway in SLE T cells contributes to the DNA hypomethylation that is a characteristic feature of SLE T cells that has also been reported in cells extracted from thymus and axillary lymph nodes of 20-week-old MRL/lpr mice (Quddus et al., 1993; Mizugaki et al., 1997). Indeed, in mature T cells, DNA methylation is controlled by the DNA methyltransferase 1, which is itself regulated by signals depending on the ERK pathway (Gorelik and Richardson, 2010; Sunahori et al., 2013). It is also noticeable that B56*a,* found here to be overexpressed in MRL/lpr splenocytes, normally leads to the dephosphorylation of B-cell lymphoma 2 (Bcl2) protein, the phosphorylation of which at Ser^70^ is required for its anti-apoptotic function. This overexpression of B56a might inhibit the activity of Bcl2 and as a matter of consequence, for instance, might increase ceramide-induced apoptosis.

Investigations performed by different groups demonstrated variable results regarding possible changes in the expression levels of CXCL12 and CXCR4 in lupus. A Japanese study showed that compared to patients with inactive lupus disease and controls, the expression level of CXCR4 on circulating B cells was significantly higher in individuals with active disease (and CXCR4-expressing B cells more frequently observed in the renal biopsy specimens) while serum levels of CXCL12 remained unchanged in these two cohorts (Hanaoka et al., 2015). These results confirmed former studies indicating that compared to controls, CD19^+^ B cells and also CD4^+^ T cells of lupus patients display increased CXCR4 expression levels, notably in neuropsychiatric lupus, that were correlated with SLEDAI score (Wang et al., 2010; Launay et al., 2013; Barrera-Vargas et al., 2018). However these data contradicted some findings dealing with CXCL12 expression, which was found to be up-regulated in tubules and glomeruli of patients with lupus nephritis (Wang et al., 2010). In an independent study including Mexican Mestizos patients with lupus, a significant defect in CXCR4 expression was detected at the surface of naive and antibody-secreting B cells, associated with an abnormal intracellular localization of the receptor (Biajoux et al., 2012). CXCR7 also displayed abnormal localization in cytosolic compartments of B cells. Lupus disease activity did not impact these expression patterns. In a polish study including 61 lupus patients (66% of them were under immunosuppressive treatment with steroid and/or cytotoxic agents during the study), the serum level of CXCL12 was statistically higher in patients compared to healthy subjects but no correlation was found according to the activity of the disease (Robak et al., 2007). A polymorphism at position 801 in the 3′-untranslated region of the CXCL12 transcript that could be present or absent in certain groups of patients, may explain some of these conflicting results and notably the amount of CXCL12 that is produced. This polymorphism (transition from G to A at this position) has been reported in association with diverse autoimmune diseases, including type 1-diabetes, systemic sclerosis and also certain groups of SLE patients (Yousry et al., 2015). A significant correlation between the SDF-1-3′G801A genotype and several typical clinical and biological features of lupus disease (photosensitivity, glomerulonephritis, serositis, vasculitis, anticardiolipin antibodies) has been reported in SLE patients. The discrepancies between the published results related to the measurement of CXCL12 levels could also be due to the integrity of CXCL12 (full length or fragment) that circulates in the peripheral blood of patients (Baerts et al., 2015).

As regard to CXCL12 biology alterations, few reports only explored relevant animal models mimicking lupus disease and the effect of therapeutic strategies focused on the CXCL12/CXCR4 axis. In the (NZBxNZW)F1 lupus mouse model, CXCL12 is produced *in situ* in the inflamed glomeruli, podocytes and to a lesser extent, endothelial and mesangial cells produce large amounts of CXCL12 (Balabanian et al., 2003). It has been described that peritoneal (Mac-1^+^CD5^+^) B1a cells that accumulate in the peritoneal cavity of normal mice under the effect of CXCL12 are hypersensitive to CXCL12 in (NZBxNZW)F1 lupus mice (de la Varga Martínez et al., 2017). This elevated sensitivity seems to be due to the NZB genetic background. The administration early in life of a mouse monoclonal antibody that displays CXCL12 antagonist activity prevented the development of autoantibodies, nephritis, and death in (NZBxNZW)F1 mice. Later in life, this treatment inhibited autoantibody production, abolished proteinuria and immunoglobulin deposition, and reversed morphological changes in the kidneys. In an independent extensive study (Wang et al., 2009), a robust increase in CXCL12 expression in the nephritic kidneys of B6.*Sle1Yaa*, BXSB and MRL/lpr mice was observed compared to control mice (measured by immunohistochemistry and ELISA). The authors demonstrated that in mice with active nephritis, CXCR4 was significantly upregulated on monocytes, neutrophils, B cell subsets, and plasma cells. This overexpression was particularly visible on the MRL/lpr B220^+^ cell fraction that contains B cells but also DN T cells that invade this mouse strain while the illness progresses with time. In the B6.*Sle1Yaa* model, the CXCR4 peptide inhibitor CTCE-9908 was shown to be very potent at reducing cellular and clinical lupus-linked failures (Wang et al., 2009). Treating (NZBxNZW) F1 mice with AMD3100 alone or in combination with the proteasome inhibitor bortezomid also showed some promise (Cheng et al., 2018). Having in hands a specific blocking agent of CXCL12, the LIT-927 molecule described here, represents therefore an invaluable advantage as it permits to investigate much more deeply the specific events taking place when the CXCL12/CXCR4 pathway is hyperstimulated, and allows specific intervention without directly interfering with the receptor itself at the surface of target cells.

CXCL12 blocking with the novel, safe, and highly selective inhibitor LIT-927 was effective in vivo, in sick MRL/lpr mice. After injection into the blood stream of outbred healthy mice, LIT was distributed throughout the body and was detectable in many organs and body fluids, primarily in the lung and kidney, two organs of importance in lupus, and in plasma. We will need to check whether this biodistribution is similar in young (healthy) and old (sick) MRL/lpr mice. Upon administration into lupus mice, LIT-927 was very efficient at decreasing the abnormally raised number of peripheral WBCs that especially invade the peripheral blood of MRL/lpr mice with time (as also several lymphoid organs). This effect might result from the fact that it readily blocks CXCL12, a chemokine that with CCL17 and CCL22, attracts Th2-type cells as well as inflammatory cells such as eosinophils on the site of inflammation (Bachelerie, 2010; Chung et al., 2017). LIT-927 modified the CXCR4 signaling pathway in splenic CD4^+^ T cells from these mice, in targeting especially the AKT-GSK3b signaling pathway, and decreased the expression of activation markers on lymphocytes, which are overactivated in vivo. Apparently LIT-927 had no correcting effect on the ERK1/2 pathway, especially on the expression of P-ERK discussed above. Finding that P-AKT(Ser^473^) expression was induced by LIT-927 is an important observation in the context of lupus as phosphorylation of AKT at this residue leads to its activation (the phosphorylation of AKT at position Ser^473^ is catalyzed by MTORC2). As P-AKT is known to regulate MTOR, this protein known to be closely related to the pathogenesis of SLE (He et al., 2020) (MTORC1 is activated and MTORC2 reduced in human lupus), was also tested. No conclusion could be raised, however, as MTOR and P-MTOR (Ser^2481^) were hardly detectable in our samples. Finally, the enhanced expression of (inactive) P-GSK3b (Ser^9^) visualized after LIT-927 treatment might also have advantageous effects on the development of the lupus disease. It may influence the many functions played by active GSK3b, notably on T cell activation, cell survival/apoptosis, and cellular responsiveness (Hoffmeister et al., 2020).

*In vitro,* LIT-927 induced a potentially beneficial decrease of expression of activation markers CD86 and CD25 on CD4^+^ T cells and of CD40 on B cells collected from the spleen of sick mice. CD25, the α-chain of IL-2 receptor, is a key component of the immune cascade of activation since when expressed with the β-chain, the receptor acquires high affinity for IL-2. CD25 and CD71 (the transferrin receptor), or costimulatory molecules, such as CD154/CD40L (a type II transmembrane protein belonging to the tumor necrosis factor family) are predominantly expressed at the surface of mature, activated lupus CD4^+^ T cells that proliferate. CD40 is a costimulatory protein found on antigen-presenting cells (among which are especially B cells in lupus). Data generated *in vitro* suggest also that LIT-927 exerts an effect on some splenic mature MRL/lpr Tregs and Treg precursors subsets. At this stage, however, it is not known if the defective functionality we previously described in MRL/lpr mice (Parietti et al., 2008) was recovered. More extensive investigations are needed to precisely define which splenic and peripheral cell types are especially rescued upon LIT-927 treatment of MRL/lpr mice and if more than the frequency of marker expression, their proper functionality is restored (Parietti et al., 2008).

## Conclusion

To conclude, our present results suggest that by hampering the interaction between CXCL12 and CXCR4, the LIT-927 neutraligand limits the signaling occurring into T and B cells in lupus context with potential favorable outcome on the course of the lupus disease that needs further investigations. Its mode of action and regulatory functions is very different from other molecules currently evaluated in advanced clinical trials. By cascade effects, down-regulating CD4^+^ T cell activation could reduce the over-activation level of lymphocytes and the autoreactive B cell differentiation into plasmocytes, which generate pathogenic autoantibodies that deposit in targeted tissues and organs and induce tissue damages. This conclusion is supported by the effect of LIT-927 on the peripheral hypercellularity that is a typical feature of the MRL/lpr mouse model that appears with time concomitantly to splenomegaly in the peripheral blood of these mice. Upon treatment, the defective regulatory functions of some cell subsets might be recovered. LIT-927 given intravenously to recipient mice showed no toxicity in C57BL/6 and CD-1 healthy mice as well as in diseased MRL/lpr mice. Other routes of administration shall be further evaluated in lupus mice to better approach a possible noninvasive protocol for human use. This is the first study describing a neutraligand of the CXCL12 chemokine able to diminish inflammatory responses in lupus. The mode of action of LIT-927, which blocks CXCL12 without directly affecting CXCR4 is extremely attractive for therapeutic purposes since CXCR4 remains accessible and active at the cell surface for fulfilling other vital functions. In addition to highlighting the unique interest of using soluble ligands, here CXCL12, as potentially valuable drug targets (Attwood et al., 2020), the results underline the importance of the CXCL12/CXCR4 axis in lupus pathophysiology.

## Data Availability

The original contributions presented in the study are included in the article/Supplementary Material, further inquiries can be directed to the corresponding author.

## Glossary

AKT: protein kinase B
A.U.: arbitrary units
BAFF: B-cell activating factor
BlyS: B Lymphocyte Stimulator
CXCL12: C-X-C chemokine 12
CXCR4 or 7: C-X-C chemokine receptor type 4 or 7
DN: double negative
EDTA: ethylenediamine tetraacetic acid
ELISA: enzyme-linked immunosorbent assay
ERK: extracellular-signal-regulated kinase
FCS: fetal calf serum
GSK3b: glycogen synthase kinase 3 beta
GPR159: G-protein coupled receptor 159
HEPES: 2-[4-(2-hydroxyethyl)piperazin-1-yl]ethanesulfonic acid
HPβCD: (2-hydroxypropyl)-β-cyclodextrin
Ig: immunoglobulin
IL: interleukin
lpr: lymphoproliferation
MEK1 or MAP/ERK: kinase-1
mitogen-activated protein kinase
MRL: Murphy Roths large
P-: phosphorylated
MTOR: mammalian target of rapamycin
PBS: phosphate-buffered saline
PP2A: protein phosphatase 2A
RA: rheumatoid arthritis
RIPA: radioimmunoprecipitation assay
SLE: systemic lupus erythematosus
SD: standard deviation
SEM: standard error of the mean
SDF-1α: cell-derived factor-1 alpha
SDS-PAGE: sodium-dodecylsulfate polyacrylamide gel electrophoresis
Tregs: regulatory T cells
WBC: white blood cells
