# Supplementary figures and images for: A Selective Neutraligand for CXCL12/SDF-1α With Beneficial Regulatory Functions in MRL/Lpr Lupus Prone Mice
Source: Front Pharmacol. 2021 Oct 21;12:752194. doi: 10.3389/fphar.2021.752194 (PMC8566942; doi:10.3389/fphar.2021.752194)

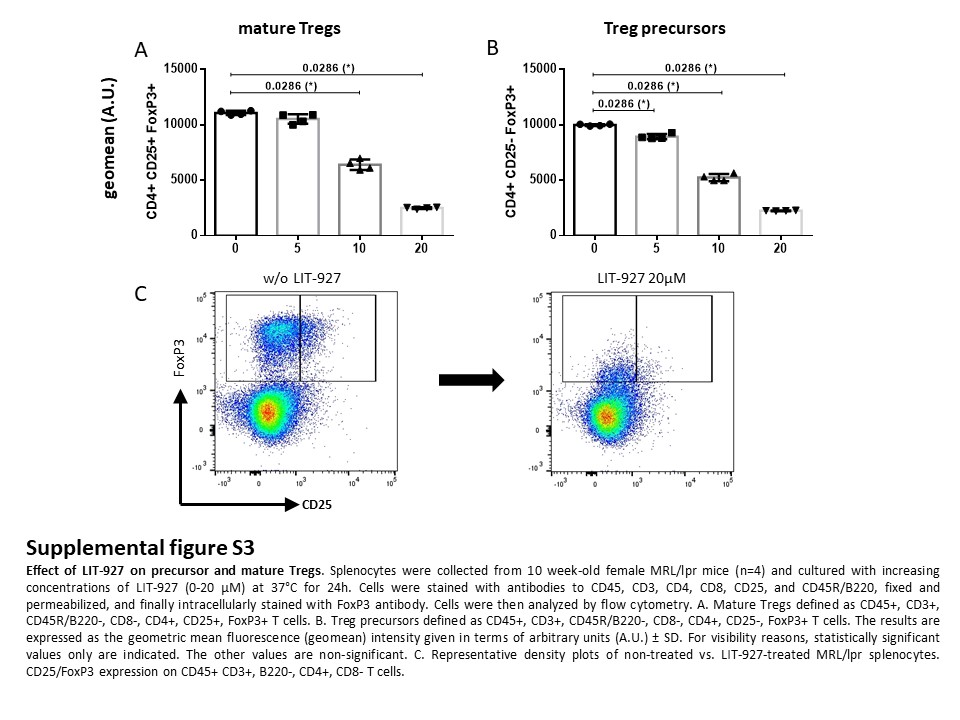

Supplement: Supplementary file 1 [file Image3.JPEG]

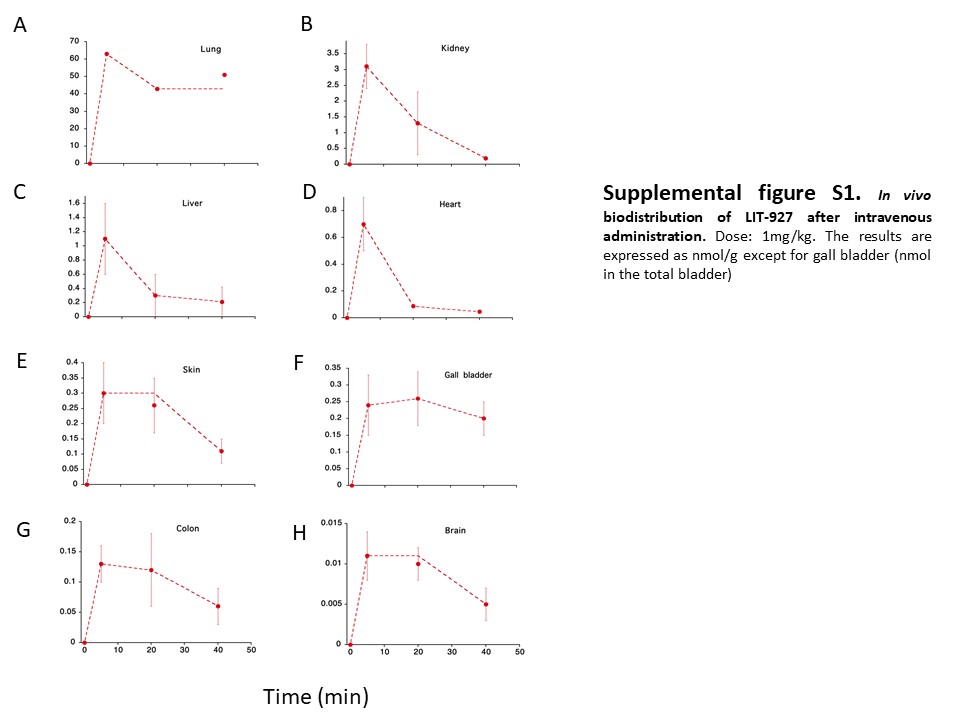

Supplement: Supplementary file 2 [file Image1.JPEG]

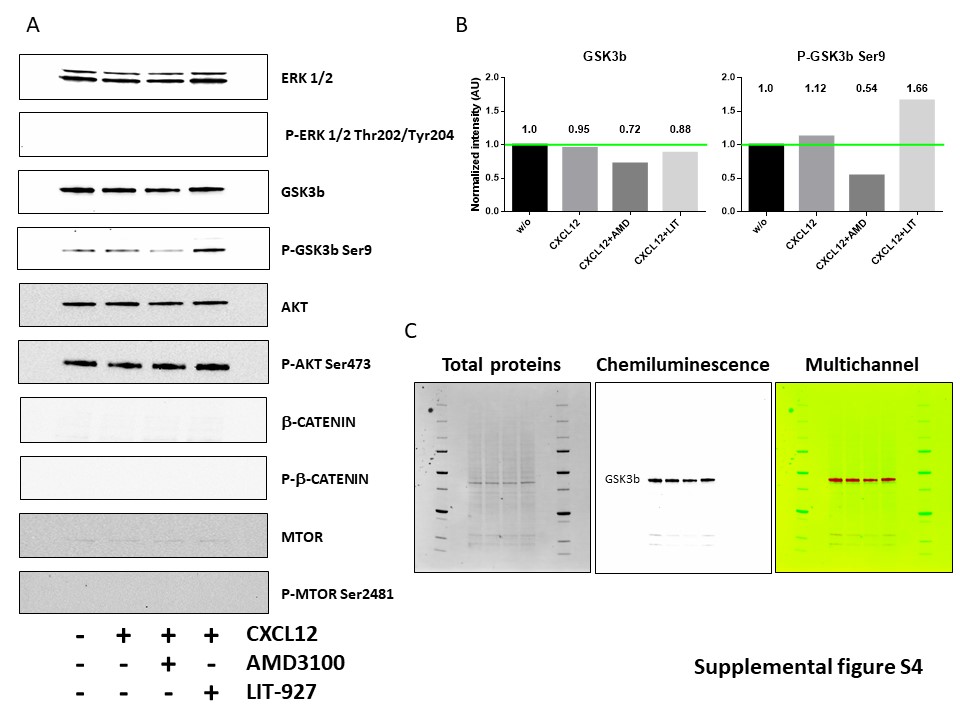

Supplement: Supplementary file 3 [file Image4.JPEG]

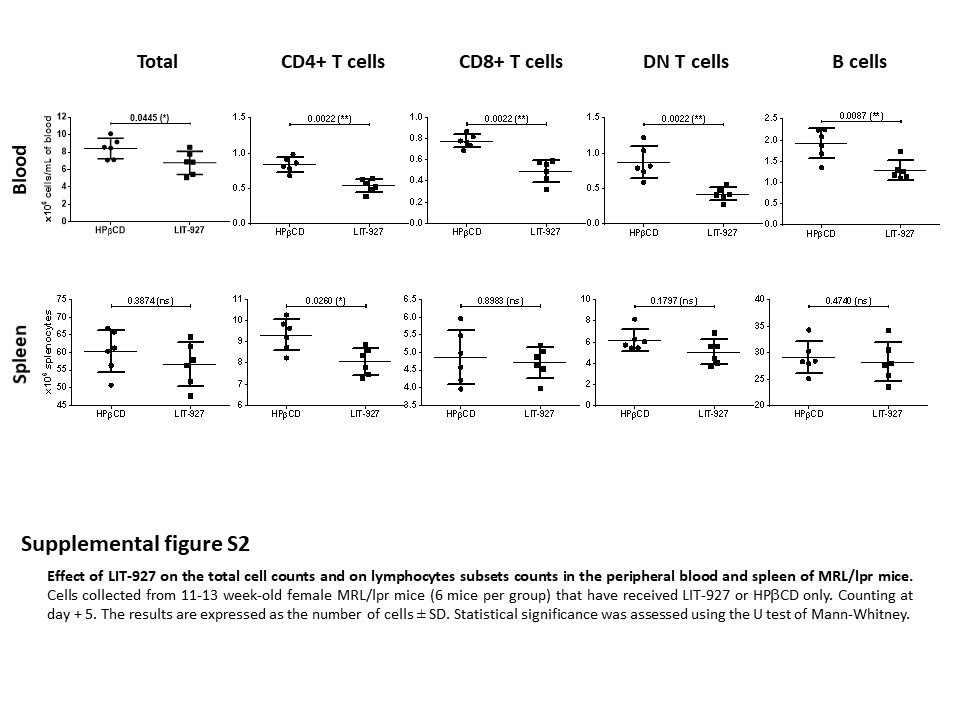

Supplement: Supplementary file 4 [file Image2.JPEG]

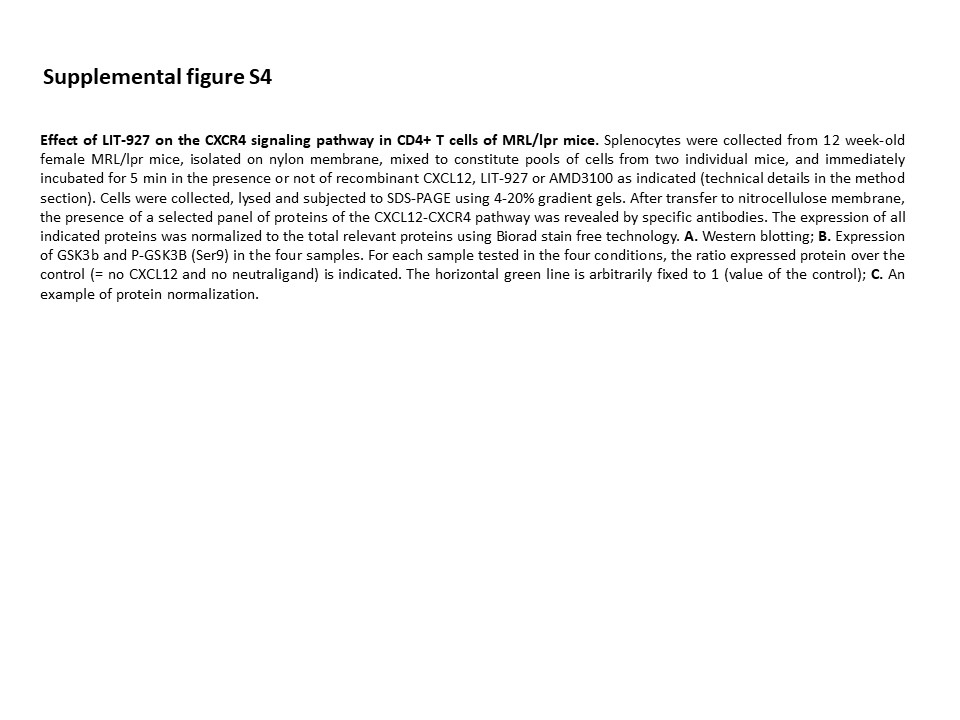

Supplement: Supplementary file 5 [file Image5.JPEG]
