# Supplementary material for: A Selective Neutraligand for CXCL12/SDF-1α With Beneficial Regulatory Functions in MRL/Lpr Lupus Prone Mice
Source: Front Pharmacol. 2021 Oct 21;12:752194. doi: 10.3389/fphar.2021.752194 (PMC8566942; doi:10.3389/fphar.2021.752194)
Supplement: Supplementary file 6 [file DataSheet1.PDF]

Supplemental Table S1: Comparison of protein expression levels in MRL/lpr and CBA/J splenocytes[a]

		Sign of change[b]	Fold change (MRL/lpr over CBA/J)[c]
**Group 1**	**Changes greater than 1.25 fold in 9 of 9 comparisons**		
PP2A reg B56α	B regulatory subunit of PP2A phosphatase	+	10.9±0.9
Janusin	Involved in interaction between neurones and glial cells	-	5.7±3.7
IGPT	Inducibly expressed GTPase, regulated by IFNγ	+	5.1±3.4
KAP	cdk associated phosphatase, may play a role in cell cycle control	-	4.8±4.0
GADS/Mona	SH domain-containing adaptator proteins	-	4.7±1.4
P115	Involved in transport from endoplasmic reticulum to the Golgi	-	4.1±1.7
Nucleoporin p62	Involved in proteins and RNA transport into and out the nucleus	-	3.9±1.5
IP3R-3	Type III receptor for Inositol triphosphate	-	3.8±1.5
Clathrin heavy chain	Involved in receptor-mediated endocytosis	-	3.8±2.3
RSer8	Involved in membrane trafficking in the presynaptic nerve terminal	-	3.2±1.7
BRAMP2	Brain form of amphiphysin 2	-	3.0±1.1
MEK1	MAPK/ERK kinase 1	-	2.9±0.4
PP2A catalytic	Catalytic subunit of PP2A phosphatase	-	2.6±0.6
RACK1	Receptor for activated C-kinase, binds to protein kinase C	-	2.5±0.7
MEK2	MAP kinase kinase 2, phophorylates ERK at Tyr/Thr residues	-	2.5±1.0
DYRK	Ser/Thr/Tyr kinase	-	2.4±0.2
BiP	Chaperon protein in the endoplasmic reticulum	-	2.4±0.6
PARP	Poly(ADP-ribose) polymerase	-	2.4±0.6
HPrp17	Non-snRNP protein important for pre-mRNA splicing	-	2.4±1.0
Caspase-3	Apoptotic protein, member of the family of cysteine protease	+	2.3±0.4
RECK	Reversion-inducing-cysteine-rich protein	-	2.3±0.5
Gα	α subunit of GTP binding regulatory proteins	-	2.3±0.7
PBCβ	Ser/Thr kinase, β isoform	-	2.2±0.7
Transportin	Involved in the export of RNA from the nucleus	-	2.1±0.4
Bid	Pro-apoptotic protein, member of the Bcl-2 family	+	2.1±0.4
p36	Involved in the regulation of cyclin-dependent kinase activation	-	1.9±0.4
Rho	Member of the Ras superfamily of GTPases	-	1.7±0.2
plCln	Component of a nucleotide-sensitive chloride channel	-	1.7±0.2
PP1	Protein phosphatase 1, Ser/Thr phosphatase	+	1.7±0.3
Cyclin D3	Regulatory subunit for cyclin-dependent kinase, associates with cdk4	-	1.6±0.2
CHD3	Mi-2 autoantigen, facilitates deacetylation of histones in vitro	-	1.6±0.3
B2 Bradykinin R	B2 subtype receptor for bradykinin	-	1.5±0.1
ERK2	Extracellular-signal-regulated kinase 2	-	1.5±0.2

Group 2	Changes greater than 1.25 fold in all 9 comparisons involving proteins migrating in the vicinity of Ig heavy/light chains		
HILP/XIAP	Human inhibitor of apoptosis protein-like protein	+	52.2±39.7
Annexin XI	$Ca2^{+}$ and phospholipid-binding protein	+	38.0±25.4
SNAP	Soluble N-ethyl-maleimide-sensitive factor attachment proteins	+	34.1±23.8
AP50	Medium chain of adaptator protein AP-2	+	9.9±5.4
cdc25B	Tyrosine phosphatase that dephosphorylates cdc2	+	9.8±5.7
PDI	Protein disulfide isomerase, multifunctional protein	+	8.4±7.0
ElF 5	Eukaryotic initiation factor 5	+	7.0±3.5
TCBP49	Taipoxin-associated calcium binding protein 49	+	6.6±4.2
Ets-1	Proto-oncogene, member of the transcription factor family	+	6.5±2.0
RAP30	RNA polymerase II-associated protein	+	6.4±1.6
LCB1	Involved in sphingolipid biosynthesis	+	5.6±2.4
14-3-3-ε	Family of proteins involved in many cellular processes	+	4.9±0.6
PP5/PPT	Ser/Thr phosphatase 5	+	4.9±1.9
Mxi-1	Max1-binding protein, regulates the oncogene Myc activity	-	4.5±1.2
Hic-5	Binds to focal-adhesion kinases	+	4.4±2.7
N-copine	C2 domain-containing phospholipid-binding proteins	+	4.1±1.7
CaMKinase IIβ	Type II Ca2+/Calmodulin (CaM)–dependent kinase	+	4.0±1.5
ILK	Integrin-linked kinase, Ser/Thr kinase	+	3.9±1.3
PKA RIIβ	Regulatory subunit IIβ of cAMP-dependent protein kinase A	+	3.6±1.4
Lyn	Cytosolic tyrosine kinase that belongs to the Src family	+	3.5±1.4
P47A	Medium chain of the neuronal AP-3 adaptator protein complex	+	3.4±0.9
ALDH	Aldehyde dehydrogenase	+	3.0±0.4
SHC	Adaptator protein, role in cell metabolism, growth and differentiation	+	3.0±0.3
PKA RI	Regulatory subunit I of cAMP-dependent protein kinase A	+	2.1±0.6
Rab27	Member of the low molecular weigh GTPases	-	2.0±0.6

[a]The proteins of interest are classified according to the general trend of protein changes and SD values taking into account intra- and inter-map reproducibility are shown. A series of proteins has been listed in a separate subset (group 2) because they co-migrate near immunoglobulin heavy or light chains, which are more abundant in MRL/lpr splenocytes compared to normal splenocytes, and can perturb the quantitative evaluation of signals corresponding to other proteins present in this region of the blot.
[b]Indicates an increase (+) or a decrease (-) in protein level expression in MRL/lpr splenocytes relative to CBA/J splenocytes.
[c]Semi-quantitative value that represents the protein changes for MRL/lpr mice relative to CBA/J mice.
